# Network pharmacology and multi-omics validation of the Jianpi-Yishen formula in the treatment of chronic kidney disease

**DOI:** 10.3389/fimmu.2024.1512519

**Published:** 2025-01-14

**Authors:** Yuyan Li, Yueming Luo, Yilan Hu, Siting Li, Guandong Li, Wanyangchuan Zhang, Xiufen Gu, Jianting Wang, Shunmin Li, Hong Cheng

**Affiliations:** ^1^ Department of Nephrology, Shenzhen Traditional Chinese Medicine Hospital, The Fourth Clinical Medical College of Guangzhou University of Chinese Medicine, Shenzhen, China; ^2^ Department of Geriatrics, Shenzhen Traditional Chinese Medicine Hospital, The Fourth Clinical Medical College of Guangzhou University of Chinese Medicine, Shenzhen, China; ^3^ The Fourth Clinical Medical College, Guangzhou University of Chinese Medicine, Shenzhen, China; ^4^ Beijing Tongrentang Hospital of Traditional Chinese Medicine, Beijing, China; ^5^ Department of Minimally Invasive Intervention and Vascular Surgery, Chongqing Red Cross Hospital (People’s Hospital of Jiangbei District), Chongqing, China

**Keywords:** Jianpi-Yishen formula, chronic kidney disease, network pharmacology, macrophage polarization, multi-omics

## Abstract

**Objective:**

Chronic kidney disease (CKD) is a major global health problem. In clinical practice, the Chinese patent herbal medicine Jianpi-Yishen (JPYS) formula is commonly used to treat CKD. However, the molecular mechanisms by which JPYS targets and modulates the host immune response remain unclear.

**Methods:**

This study utilized network pharmacology, RNA sequencing (RNA-seq), and metabolic analyses using *in vivo* and *in vitro* models to investigate the impact of the JPYS formula on inflammation and the immune system. Specifically, the study focused on macrophage polarization and metabolic changes that may slow down the progression of CKD.

**Results:**

A total of 14,946 CKD-related targets were identified from the GeneCards and Online Mendelian Inheritance in Man (OMIM) databases through network pharmacology analyses. 227 potential targets of the JPYS formula were predicted using the TCMSP database. Additionally, network diagram demonstrated that 11 targets were associated with macrophage activity. *In vivo* studies indicated that the JPYS formula could reduce blood urea nitrogen and serum creatinine in adenine-induced CKD rats. Furthermore, the formula inhibited inflammatory damage and abnormal macrophage infiltration in this CKD model. RNA-seq, proteomic and metabolic analyses identified the regulation of amino acid metabolism by betaine, specifically referring to glycine, serine, and threonine metabolism, as a key target of the JPYS formula in slowing the progression of CKD. In addition, *in vitro* studies suggested that JPYS may enhance tryptophan metabolism in M1 macrophage polarization and betaine metabolism in M2 macrophage polarization.

**Conclusions:**

The JPYS formula has been shown to have beneficial impact on CKD; a key mechanism is the mitigation of inflammatory damage through the interaction between amino acid metabolism and macrophage polarization. Of specific importance in this context are the roles of tryptophan in M1 polarization and betaine in M2 polarization.

## Graphical Abstract

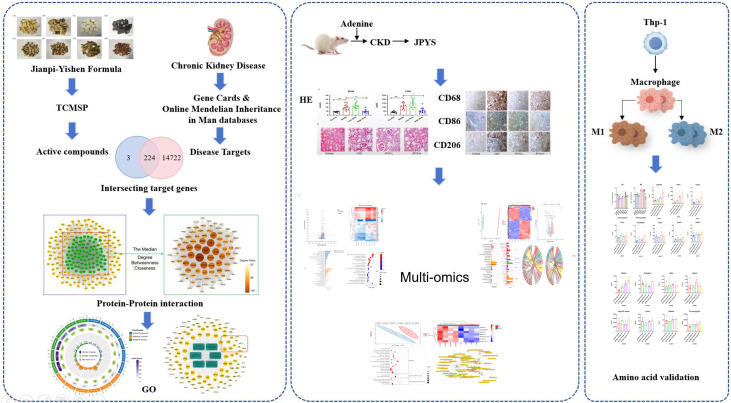

## 1 Introduction

Chronic kidney disease (CKD) is characterized by long-lasting abnormalities in renal structure or function. These abnormalities last longer than three months and have serious health repercussions (1). The global prevalence of CKD is estimated to be approximately 10–14% (1). Furthermore, CKD accelerates the aging process and promotes the development of end-stage renal disease. This leads to increased disability, decreased life expectancy, and a high annual mortality rate, all of which are important contributors to the worldwide burden of disease (2, 3). However, there are no specific treatment modalities available that can entirely arrest the progression of CKD, and coping with CKD poses challenges for both patients and their caregivers (4).

Chinese herbal medicines (CHMs) are characterized by their intricate chemical compositions, which complicates the process of identifying the specific constituents that collectively contribute to the therapeutic effects of these herbal remedies, as these are typically applied in a multi-ingredient manner (5). Previous studies have demonstrated that substances originating from Chinese herbal remedies can ameliorate CKD via multiple molecular pathways (6–9). In addition, certain formulations, such as the Sanqi oral solution and the Bupi Yishen formula, have demonstrated positive impact on kidney function (10, 11). Herbal medicine also has numerous advantages over chemical agents in the management of CKD, not least because of its diverse ingredients (12). The JPYS formula, which translates to “strengthen the spleen and kidney”, is a patented traditional Chinese medicine (TCM) formulation developed by Professor Li Shunmin, a distinguished physician of traditional Chinese medicine in Guangdong Province, drawing upon decades of comprehensive clinical experience. In previous research, the effectiveness and safety of the JPYS formula in CKD patients have been investigated (13). More recently, there are randomized controlled trials being conducted to further explore its clinical application. Previous studies have identified multi targeted effects of the JPYS formula in slowing the progression of CKD, including anti-inflammatory properties, protection against iron deficiency anemia (14), inhibition of mitochondrial fission, promotion of mitochondrial fusion, and suppression of oxidative stress, among others (15). Notably, the anti-inflammatory effects of the JPYS formula have been recognized as therapeutically significant (16) . However, the specific molecular mechanisms through which the JPYS formula targets the endogenous immune response remain unclear.

Macrophages play essential roles in immune surveillance and the maintenance of kidney homeostasis (17). Throughout the progression of CKD, macrophage polarization has been implicated in the mechanisms of inflammatory injury, renal interstitial fibrosis, and kidney repair (18). Various stimuli can influence the functional phenotype of macrophages, leading to the differentiation towards classically activated macrophages (M1) or alternatively activated macrophages (M2). M1 macrophages are recognized as pro-inflammatory cells that contribute to the progression of kidney injury, whereas M2 macrophages are traditionally known as anti-inflammatory cells. Some TCM therapies, encompassing both formulated remedies and herbal active ingredients, have demonstrated efficacy in modulating macrophage polarization towards either M1 or M2 phenotypes in kidney disease (19, 20). As such, the immunoregulatory properties of herbal medicine, particularly its anti-inflammatory effects, present a novel approach for the management of kidney diseases (21).

In this study, network pharmacology, RNA sequencing (RNA-seq), proteomics and metabolic analyses were employed to examine the impact of the JPYS formula on inflammation, the immune response, macrophage polarization, and metabolic changes that may inhibit the progression of CKD. The findings reveal that the interaction between amino acid metabolism and macrophage polarization serves as a key mechanism through which the JPYS formula mitigates inflammatory injury in CKD.

## 2 Materials and methods

### 2.1 Network pharmacology

#### 2.1.1 Screening and prediction of effective chemical constituents in the JPYS formula

The active chemical constituents of the JPYS formula (*Astragali Radix, Atractylodis Macrocephalae Rhizoma, Dioscoreae Rhizoma, Cistanches Herba, Amomi Fructus Rotundus, Salviae Miltiorrhizae Radix et Rhizoma, Rhei Radix et Rhizoma, Glycyrrhizae Radix et Rhizoma*) were obtained from the Traditional Chinese Medicine System Pharmacology Database (old.tcmsp-e.com/tcmsp.php, updated until September 2023) (22). The criteria of oral bioavailability (OB) ≥ 30% and drug likeness (DL) ≥ 0.18 were applied to assess the active ingredients of the JPYS formula and identify the pertinent effective active ingredients. The active ingredients of the JPYS formula were then converted into the corresponding human gene names using the Uniprot database. By utilizing “chronic kidney disease” as the keyword, the GeneCards and Online Mendelian Inheritance in Man databases were utilized to retrieve genes associated with CKD. The identified CKD-related genes and drug target genes were mapped to identify the common target genes of the “JPYS formula-CKD”.

#### 2.1.2 “Drug-Ingredient-Target-Disease” visualization network construction

The active components of the JYPS formula and the common target genes of the “JYPS formula–CKD” were imported into Cytoscape 3.7.1 software for visualization. Subsequently, a network diagram of “drug–ingredient–target–disease” was then established. Each node in the diagram symbolized a disease, drug, bioactive ingredient of a drug, or target, with the connections between nodes indicating the interrelations among the disease, drug, bioactive ingredient, and target.

#### 2.1.3 Protein-protein interaction network and, gene ontology functional analyses

The shared targets of the JPYS formula and CKD were entered into the STRING database (https://string-db.org/) using specific parameters to extract the PPI network. The analysis focused on the human species (Homo sapiens) with a protein relationship score threshold of 0.4. The presence of free proteins was concealed to obtain the protein interaction network. The protein-protein interaction network data was downloaded and imported into Cytoscape 3.7.1 software. Utilizing the Network Analyzer tool, a topological analysis was conducted on the relevant parameters of drug-disease common targets, which included connectivity (Degree), betweenness centrality, and closeness centrality. Targets exceeding the median values of the aforementioned parameters were designated as core targets.

The Gene Ontology Biological Process (BP), Molecular Function (MF), and Cellular Component enrichment analysis data, were obtained from the STRING database. The GO analysis conditions were set to include observed gene count and strength both greater than the median. Subsequently, the top 6 significantly enriched items in BP, MF and CC were selected and import into ChiPlot (https://chiplot.online/) to generate a circular enrichment plot. Additionally, the top 6 significantly enriched items in GO-BP and their associated targets were imported into Cytoscape 3.7.1 software for visualization processing, resulting in the creation of a network diagram titled “BP Entry - Target.” 

### 2.2 JPYS formula preparation

Medicinal herbs for JPYS formula were gained from the Pharmaceutical Department of Shenzhen Traditional Chinese Medicine Hospital. The JPYS formula comprises the following eight herbs: *Astragali Radix, Atractylodis Macrocephalae Rhizoma, Dioscoreae Rhizoma, Cistanches Herba, Amomi Fructus Rotundus, Salviae Miltiorrhizae Radix et Rhizoma, Rhei Radix et Rhizoma, Glycyrrhizae Radix et Rhizoma.* These raw herbs were weighed and boiled twice for 1 h each time in 8 times of water. Our earlier research detailed the preparation and quality control of JPYS formula extract (15).

### 2.3 Animals and experimental treatments

Male Sprague Dawley rats(ethics approval reference number:TOP-IACUC-2021-0112) aged 6–8 weeks were randomly assigned to one of four groups: control (n = 8), CKD (n = 10), CKD + JPYS-L (n = 10), and CKD + JPYS-H (n = 10). Rats in the CKD and CKD + JPYS were fed a diet containing 0.75% adenine for 3 weeks, followed by a normal diet for 1 week. Rats in the control group were fed a normal diet for 4 weeks. The CKD + JPYS groups were administered with 5.44 g/kg/day of JPYS extract (CKD + JPYS-L, low-dose group) and 10.89 g/kg/day of JPYS extract (JPYS-H, high-dose group) via gastric irrigation for 4 weeks during the study period.

### 2.4 Biochemical analysis

Serum creatinine and urea nitrogen levels were measured using a Roche automatic biochemistry analyzer (Tokyo, Japan) in accordance with the manufacturer’s instructions.

### 2.5 Histological analysis and immunohistochemistry

Paraffin-embedded kidney tissues extracted from four groups of rats were cut into 3-µm sections, dewaxed, and rehydrated. Sections were stained with hematoxylin and eosin (H&E) stain and visualized. Immunohistochemistry was performed according to the established protocol as described previously (23). Antibodies used are in **Supplementary Table 1**.

### 2.6 RNA-seq

The kidney samples from the CKD and CKD+JPYS-H groups underwent analysis at the Beijing Genomics Institute (BGI, Shenzhen, China). The samples were purified and amplified through polymerase chain reaction (PCR). The PCR yield was quantified using Qubit, and the samples were combined to produce a single-stranded DNA circle (ssDNA circle) which generated DNA nanoballs. These nanoballs were then loaded into patterned nanoarrays. Subsequent data analysis was conducted using the BGISEQ500 platform.

### 2.7 Kidney proteomics analysis

Proteomics analysis was conducted at the Climb Technology Co., Ltd. Briefly, kidney tissue were thoroughly lysed using a protein lysis buffer, followed by the measurement of protein concentration. Based on the results of these measurements, an appropriate volume of protein was extracted from each sample for enzymatic hydrolysis. The subsequent day, the ultrafiltration tubes were centrifuged at 13000g at room temperature for 10 minutes. The liquid collected in the collectiong tube was then transferred to a new centrifuge tube and subjected to vacuum drying. The samples were subsequently desalinated utilizing a C18 desalination column.

For mass spectrometry detection, the chromatographic mobile phase A consisted of 0.1% formic acid, while phase B comprised 80% acetonitrile and 0.1% formic acid. The freeze-dried peptide segments were completely dissolved in solution A (0.1% formic acid) and centrifuged at 17,000 g for 15 minutes. The supernatant was then added to the built-in tube and placed in the automatic sampling device. The sample was introduced into the C18 analytical column (inner diameter 150 μm, 25 cm) from the automatic sampler at a flow rate of 1.2 μL/min using the EASY nLC 1200 liquid chromatography system (Thermo, USA) for elution. The elution conditions for the liquid chromatography were set at a flow rate of 600 nL/min, with the B solution (acetonitrile containing 0.1% formic acid) increasing linearly from 6% to 30% over 0 to 42 minutes, followed by a further increase from 30% to 42% between 42 and 51 minutes, and finally rising to 95% within 5 minutes, which was maintained for 60 minutes.

The Thermo Scientific Q Exactive HF mass spectrometer, equipped with a Nanospray Flex ion source, was utilized, with the ion spray voltage set to 2.3 kV and the temperature of the ion transfer tube maintained at 320°C. The mass spectrometer operated in Data-Independent Acquisition (DIA) mode. Following the collection of DIA data, the Spectronaut 18.0 software (Biognosys) was employed to search the human database downloaded from Uniprot.

### 2.8 Kidney metabolome analysis

Kidney samples from the CKD and JPYS-H groups (n=4 in each group) were processed by combining them with a standard chromatography and mass spectrometry protocol. Briefly, The procedure involved several methodical steps: (1) Kidney samples were processed through homogenization in 80% methanol and subsequently incubated at -80°C for a duration of two hours. After the incubation, the mixture was subjected to centrifugation, and the supernatant was collected and evaporated using nitrogen gas. To facilitate reconstitution, 100µl of an acetonitrile-water solution (in a 4:1 ratio) was added. The resulting mixture was vortexed, centrifuged, and the supernatant was transferred to a liquid phase vial for further analysis; (2) The mobile phase A consisted of 0.1% formic acid in ultrapure water, while mobile phase B was composed of methanol. The flow rate was maintained at 0.30 mL/min, with the oven temperature set at 40°C. The autosampler temperature was regulated at 10°C, and the injection volume was 2 µL utilizing a full loop injection method; (3) Targeted profiling was conducted using a QTRAP^®^ 5500 System (SCIEX) operating in positive mode, employing the Multiple Reaction Monitoring (MRM) technique. The electrospray ionization parameters were optimized for a flow rate of 0.30 mL/min, with the following specifications: electrospray voltage of 5500 V, temperature of 500°C, curtain gas at 40, CAD gas at 12, and gases 1 and 2 set at 50 psi each.

### 2.9 Cell culture

THP-1 cells were cultured in a 6-well plate and incubated with 10 ng/ml of Phorbol 12-myristate 13-acetate (PMA) for 48 hours. Followed by incubated in different doses of JPYS formula for 24h and 48h. Cell Counting Kit-8 (CCK-8) was used to assess cell viability.

Afterwards, the macrophages were stimulated into M1 and M2 polarization, respectively. In M1 polarization, LPS (100 ng/ml) and IFN-γ (20 ng/ml) were used to stimulate M1 macrophages. The groups were divided into different categories, including Macrophage, M1-Macrophage, and JPYS formula doses (M1 Macrophage incubated with different JPYS formula doses) for 48h. In M2 polarization, IL-4 (25ng/ml) and IL-13 (25ng/ml) were used to stimulate M2 macrophages. The groups were divided into different categories, including Macrophage, M2-Macrophage, and JPYS formula doses (M2 Macrophage incubated with different JPYS formula doses) for 48h.

### 2.10 Macrophage ultra-performance liquid chromatography tandem mass spectrometry

The methodology employed for the extraction of metabolites from cells, chemicals, reagents, and the UPLC-MS/MS conditions adhered to the procedures outlined in previous studies (24) .The protocol involved several steps: (1) Cell samples were treated with Methanol for shaking and lysis, followed by incubation at -80°C for 30 minutes. Subsequently, the samples were subjected to shaking and centrifugation, and the resulting supernatant was dried using a nitrogen blower. Prior to sample running, re-dissolution was performed. Additionally, the preparation and optimization of an amino acid standard solution were carried out, including the determination of parent and daughter ions of the standard. (2) Standard curves were created at various concentrations (1000%, 500%, 200%, 100%, 80%, 40%, 20%, 10%, 5%) along with the configuration of the mobile phase, liquid phase method, and mass spectrometry method. The samples were then analyzed using LC-MS. (3) Experimental sample concentration involved running the samples on a C18 chromatography column, with adjustments made for samples with low amino acid content. Samples that did not produce peaks were rerun using Glycan columns with corresponding adjustments to the mobile phase and mass spectrometry method. (4) Machine testing was conducted by mixing 50µl of supernatant from each sample in a centrifuge tube, followed by randomization and interspersion of Quality Control (QC) samples with cell samples. The samples were numbered and sorted based on random numbers, with QC values used to calculate Coefficient of Variation (CV) values. A CV value within 15% indicated acceptable system deviation. QC samples 1-10 were configured to control sample quality, and new standard curve ranges were established based on sample concentration test results. Post-sample run, Multi Quant software was utilized for result analysis.

### 2.11 Statistical analysis

The measurement data was presented as the mean ± SEM. The one-way analysis of variance (ANOVA) or the Kruskal-Wallis test was employed to assess significant differences among groups. Statistical analysis was conducted using GraphPad Prism software, with a significance level set at *P* < 0.05.

## 3 Results

### 3.1 Network pharmacology showed that JPYS formula might reduce CKD progression via different targets and pathways

**Figure 1:**
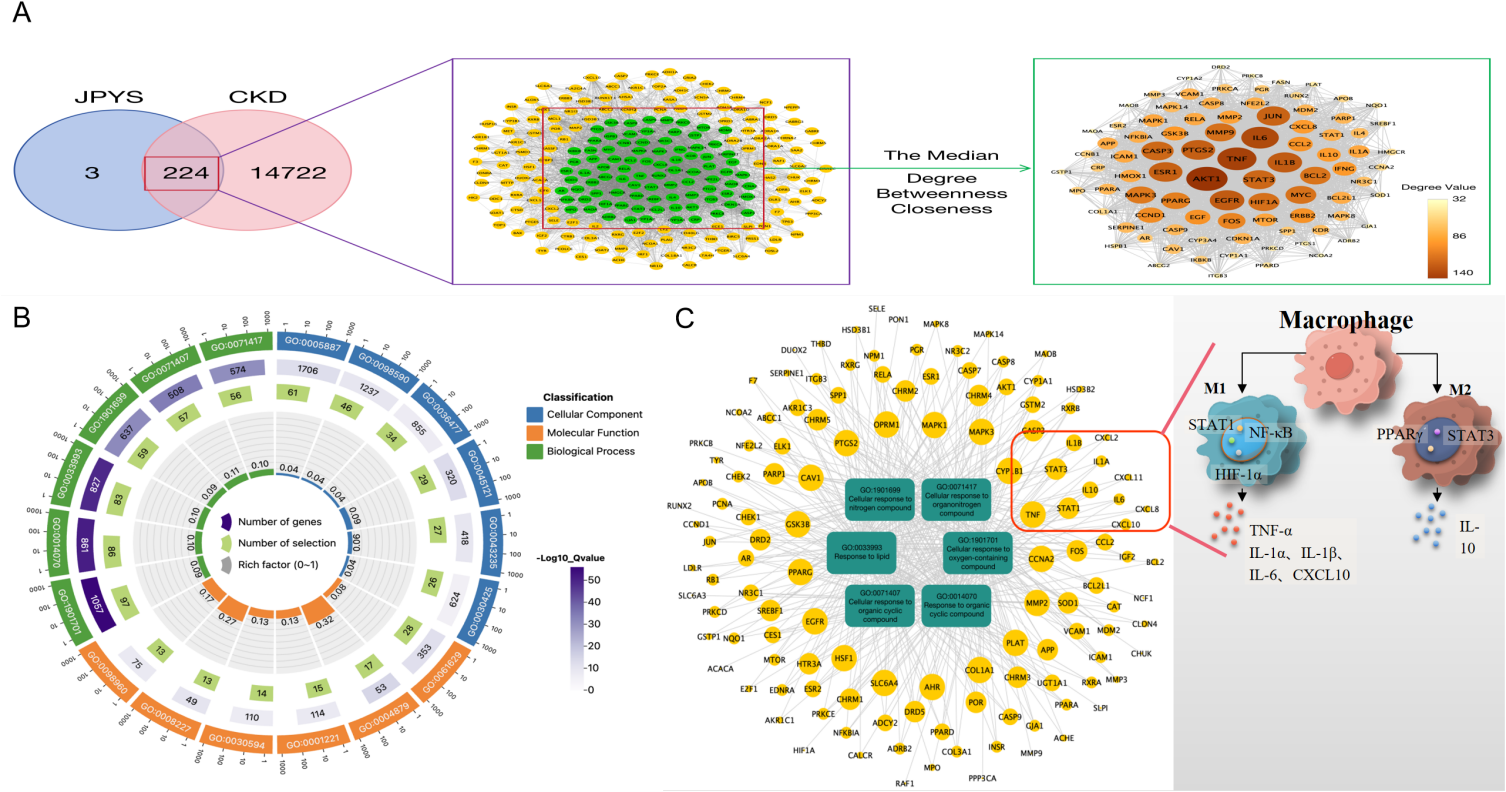
Network pharmacology screening and prediction of effective chemical constituents in the JPYS formula. **(A)** 227 potential targets of the JPYS formula were predicted by TCMSP database, and 14,946 CKD-related targets were collected via the GeneCards and OMIM databases. The comparison of the targets identified via these two methods revealed 224 overlapping targets. Core targets were considered with targets greater than the median of the above parameters, totaling88. **(B)** Top 6 significantly enriched items in BP/MF/CC; **(C)** “BP entry target” network diagram demonstrated that 11 targets were associated with macrophage activity.

933 active ingredients of the JPYS formula were retrieved through the TCMSP database and further screening performed using the parameters of OB ≥ 30% and DL ≥ 0.18 revealed 224 potential active ingredients (**Supplementary Table 2**). Afterwards, 227 potential targets of the JPYS formula were predicted by TCMSP database, and 14,946 CKD-related targets were collected via the GeneCards and OMIM databases. The comparison of the targets identified via these two methods revealed 224 overlapping targets (**Figure 1A**). Imported 224 common targets of JPYS formula and chronic kidney disease into the STRING database to obtained PPI protein interaction network data. Afterwards, the data were imported into Cytoscape 3.7.1 software to analyze and obtain the connectivity (Degree), BC and CC of drug-disease common targets. Core targets were considered with targets greater than the median of the above parameters, totaling 88 (**Figure 1A**).

### 3.2 GO analysis of “JPYS formula–CKD”

2117 GO enrichment analysis entries were obtained in the STRING database, including 1784 for BP analysis, 203 for MF analysis, and 130 for CC analysis. Set the analysis conditions to observed gene count and strength both greater than the median, and obtain the top 6 significantly enriched items in BP/MF/CC (**Figure 1B**). Establishing a “BP entry target” network diagram, it was found that 11 targets were associated with macrophage activity, as shown in **Figure 1C**.

### 3.3 JPYS formula exhibits renoprotective effects in CKD rats

**Figure 2:**
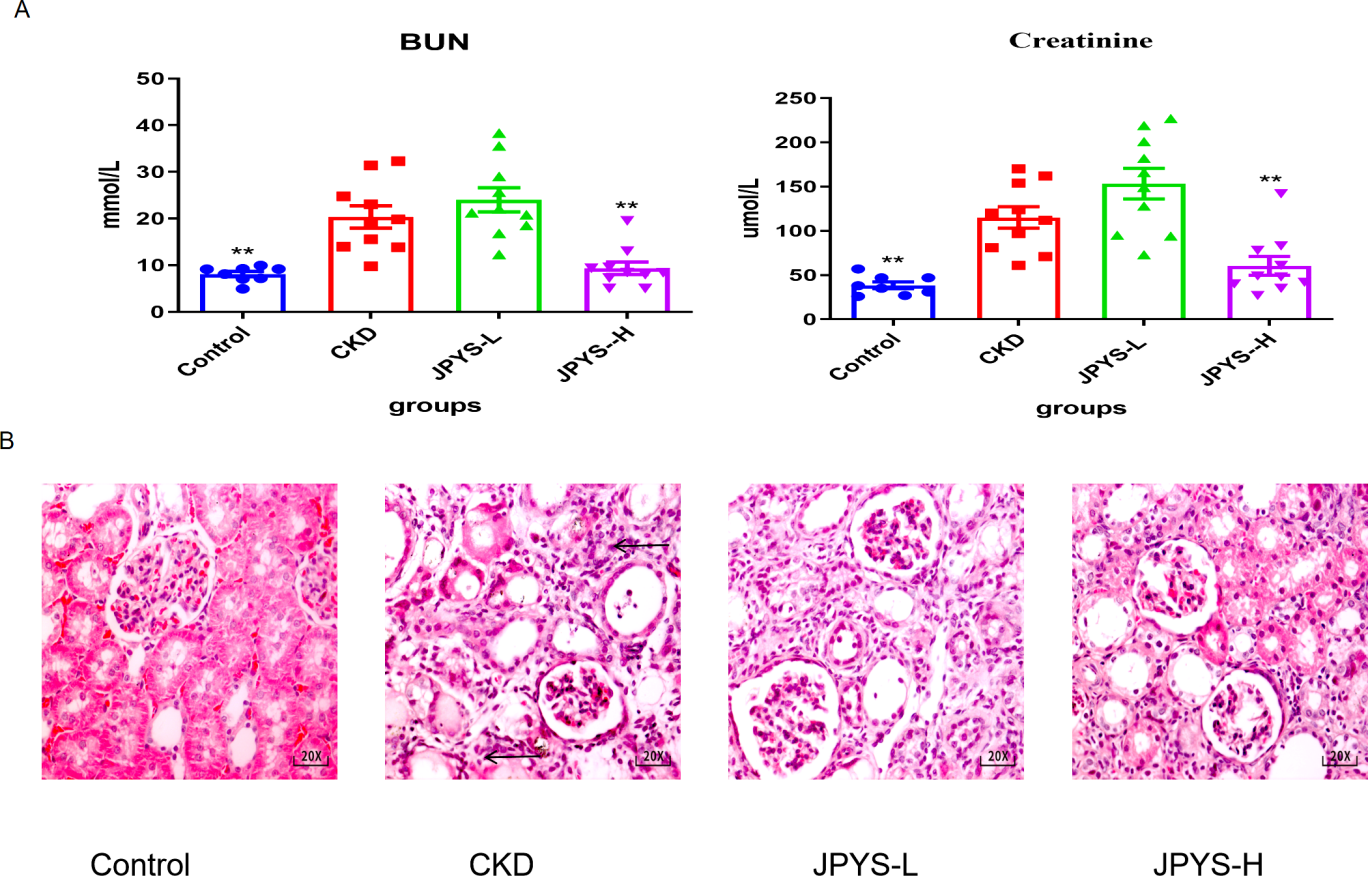
Effects of JPYS formula on renal function and pathological injury in CKD rats. **(A)** Blood urea nitrogen levels and serum creatinine levels. **(B)** Representative HE staining images in each groups. The arrows indicated the usual pathological alterations associated with CKD. **Represents a significant variation compare with the CKD group among the multiple comparisons. **P < 0.05.

Rats in the CKD group exhibited higher levels of serum creatinine (Cre) and blood urea nitrogen (BUN), which were restored after JPYS formula treatment (**Figure 2A**). H&E staining revealed the CKD group exhibited inflammatory injuries and fibrotic changes, while the CKD + JPYS group showed a significant reduction in pathological injuries, consistent with the improvement in renal function (**Figure 2B**).

**Figure 3:**
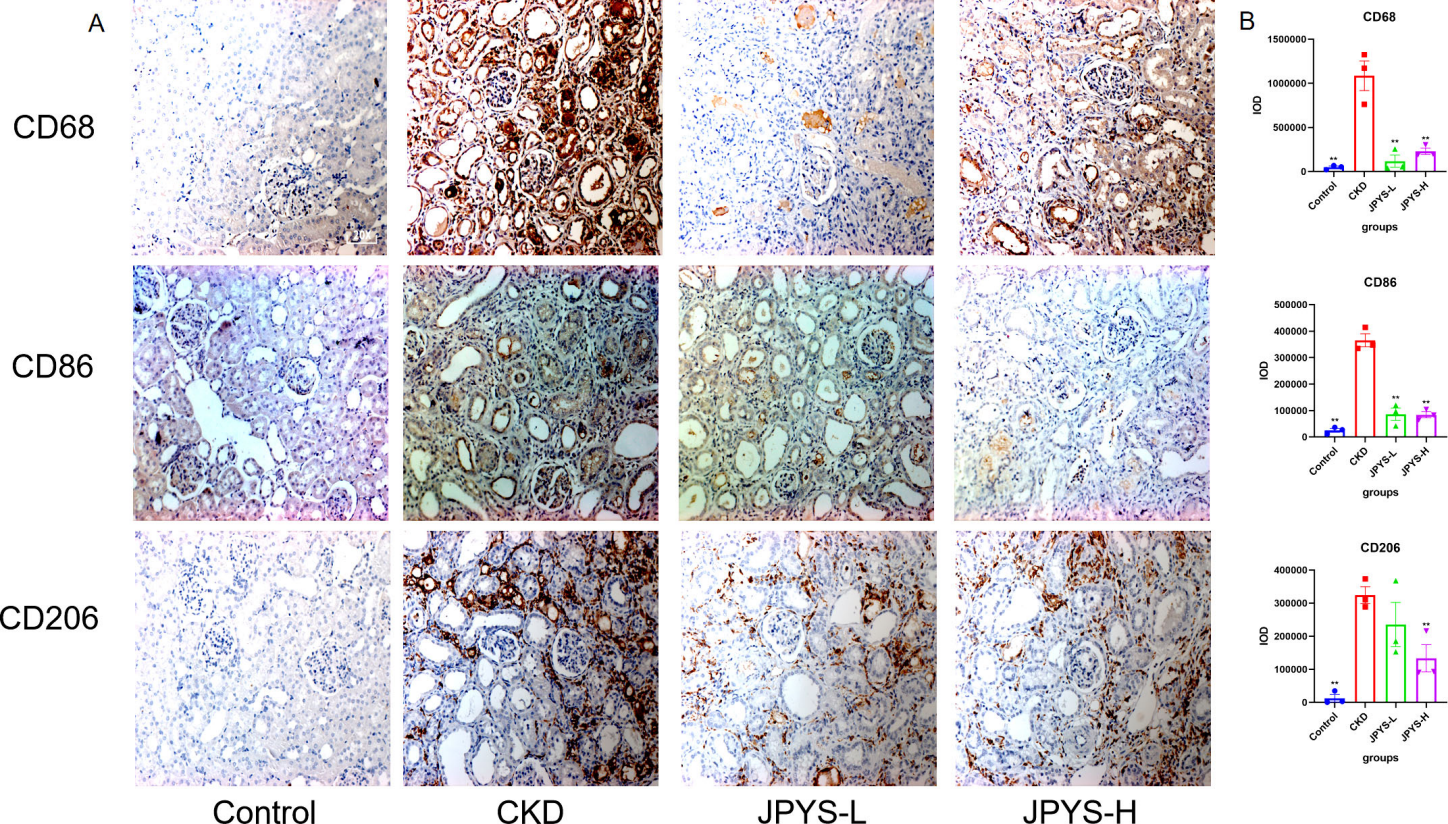
Representative immunohistochemistry images and IOD values of CD68, CD86 and CD206 expression in the kidneys of rats. **(A)** All images are shown at identical magnification, ×200. **(B)** The IOD values among various groups. **Represents a significant variation compare with the CKD group among the multiple comparisons. **P < 0.05.

To confirm whether the renoprotective effect of JPYS formula was associated with the modulation of macrophages, immunohistochemistry was performed to measure the expression of CD68 and CD86 in the kidney tissue. The CKD group exhibited higher levels of CD68 and CD86 expression than the control group, while the JPYS group showed lower expression levels than the CKD group (**Figure 3A**). Additionally, there were notable statistical differences in Integrated Optical Density (IOD) values among various groups (**Figure 3B**). These results indicate that JPYS formula therapy down-regulates macrophages, including M1 and M2 macrophages in the kidneys of CKD rats.

### 3.4 Amino acid metabolism may be the core targets for JPYS formula to delay the progression of CKD

**Figure 4:**
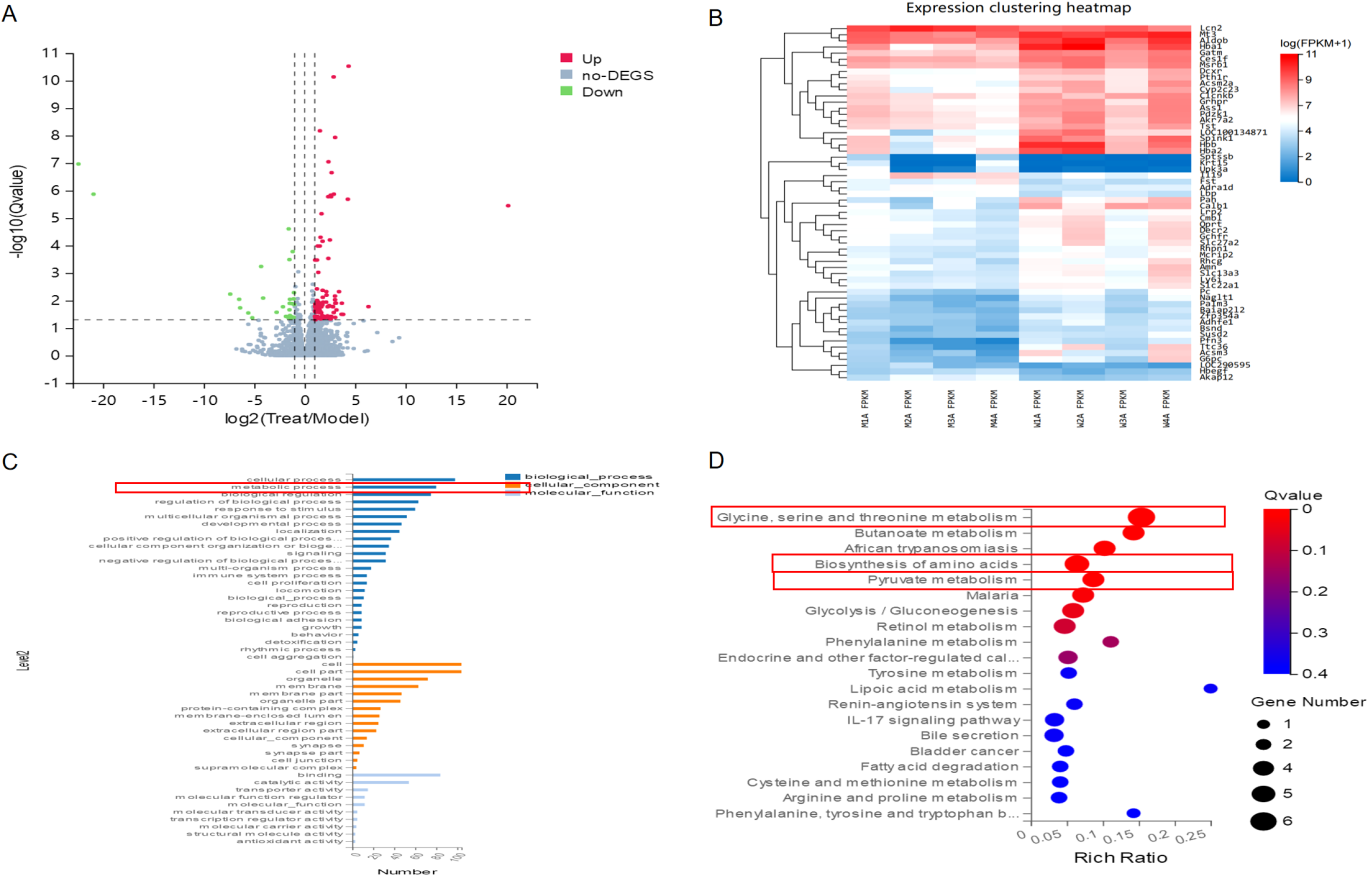
RNA-seq between CKD and JPYS groups. **(A)** Volcano plot analysis showed 132different genes between the CKD group and JPYS groups. **(B)** Heatmap showed different cluster between CKD and JPYS groups. **(C)** The cluster analysis of the GO classification revealed a significant enrichment of the metabolic process. **(D)** In the KEGG pathway, the Glycine, serine, and threonine metabolism (Amino acid metabolism), Butanoate metabolism (Carbohydrate metabolism), Biosynthesis of aminoacids (Global and overview maps), Pyruvate metabolism (Carbohydrate metabolism), and Glycolysis/Gluconeogenesis (Carbohydrate metabolism) were enriched.

RNA enrichment analysis was conducted to compare the differences between CKD and CKD+JPYS groups in RNA-seq (n = 4 per group). There’s 132 different genes between the CKD group and JPYS groups (**Figure 4A**). And the heatmap showed different cluster between CKD and JPYS groups (**Figure 4B**). The cluster analysis of the GO classification in RNA-seq revealed a significant enrichment of the metabolic process (**Figure 4C**) between CKD and JPYS formula. In the Kyoto Encyclopedia of Genes and Genomes (KEGG) pathway, the Glycine, serine, and threonine metabolism (Amino acid metabolism), Butanoate metabolism (Carbohydrate metabolism), Biosynthesis of amino acids (Global and overview maps), Pyruvate metabolism (Carbohydrate metabolism), and Glycolysis/Gluconeogenesis (Carbohydrate metabolism) were enriched. This suggests that JPYS formula adjusted the metabolic function in CKD rats (**Figure 4D**).

**Figure 5:**
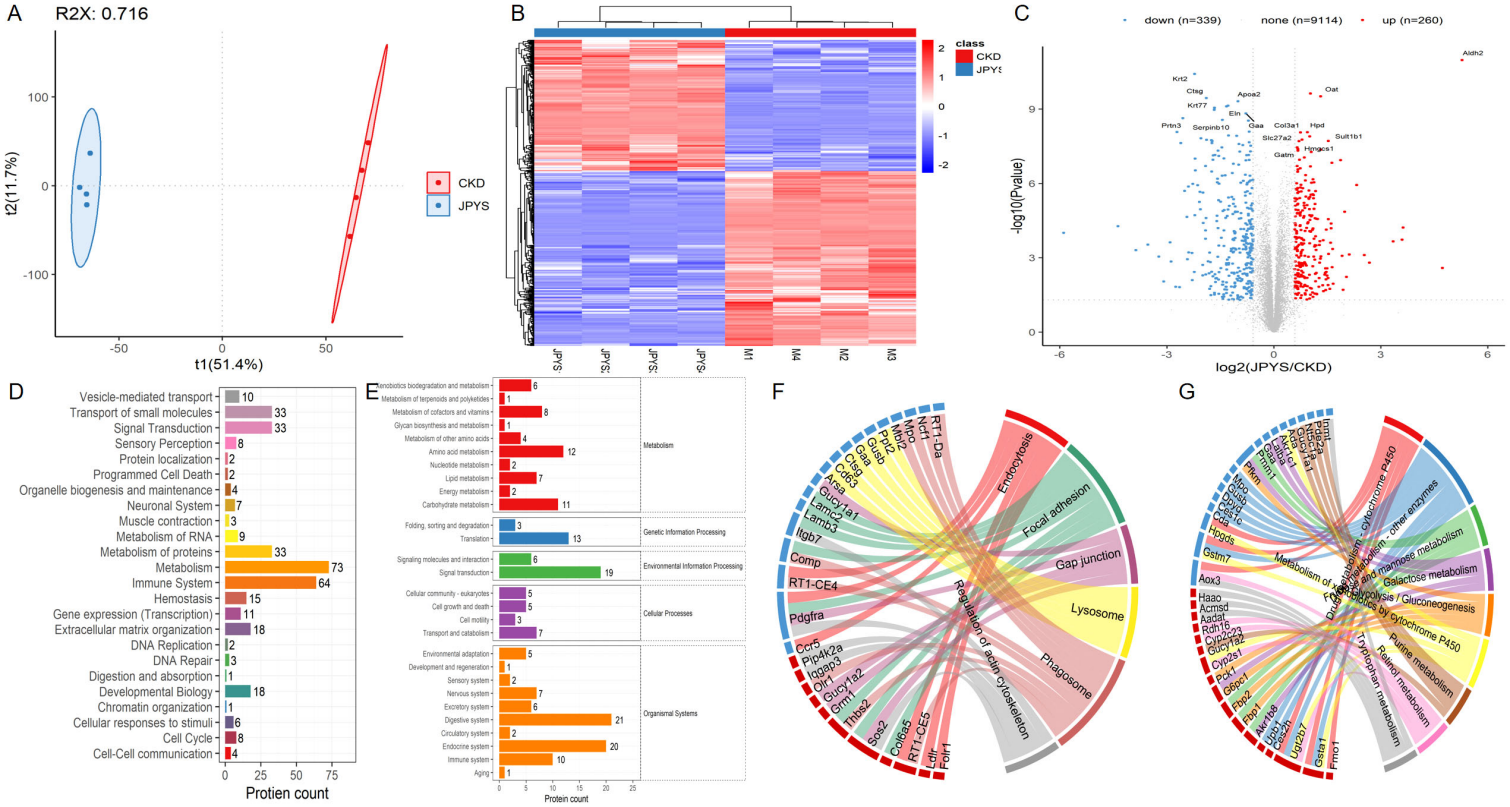
The proteomics analysis in kidney tissue. **(A, B)** The PCA and heatmap suggested that JPYS rats could be separated from CKD rats. **(C)** Volcanoplot revealed that JPYS formula exhibited up-regulated 260 proteins while down-regulated 339 proteins than CKD. **(D)** An enrichment analysis of the pathway functional entries within the Reactome database, where differential proteins were identified, indicated that the immune system and metabolic pathways were critical for interventions involving JPYS. **(E)** In metabolic pathways, the importance of amino acid metabolism, as well as the metabolism of other amino acids, played a crucial role in the process of upregulation. **(F)** A comprehensive KEGG analysis of cellular processes revealed that the regulation of the actin cytoskeleton, phagosome, and lysosome were significant biological processes influencing the differential proteins associated with JPYS. **(G)** And the tryptophan played a pivotal role in the metabolism pathway.

Moreover, the proteomics analysis, which included PCA and heatmap visualization, suggested that JPYS rats could be separated from CKD rats (**Figures 5A, B**). Volcanoplot revealed that JPYS formula exhibited up-regulated 260 proteins while down-regulated 339 proteins compared to CKD (**Figure 5C**). An enrichment analysis of the pathway functional entries within the Reactome database, where differential proteins are identified, indicates that the immune system and metabolic pathways are critical for interventions involving JPYS (**Figure 5D**). Additionally, we performed an extensive analysis of the differences in metabolic pathways through proteomics, which demonstrated that JPYS could upregulate various metabolic pathways, particularly highlighting the significance of amino acid metabolism and the metabolism of other amino acids in this upregulation (**Figure 5E**). These results further validated our transcriptomic findings. A comprehensive KEGG analysis of cellular processes revealed that the regulation of the actin cytoskeleton, phagosome, and lysosome are significant biological processes influencing the differential proteins associated with JPYS (**Figure 5F**). And the tryptophan played a pivotal role in the metabolism pathway (**Figure 5G**). This finding corroborated our previous network pharmacology hypothesis that macrophages may served as vital cellular targets for JPYS in the context of delaying CKD.

### 3.5 The regulation of amino acid metabolism by Betaine in macrophage polarization may serve as a potential target for the JPYS formula in delaying the progression of CKD

**Figure 6:**
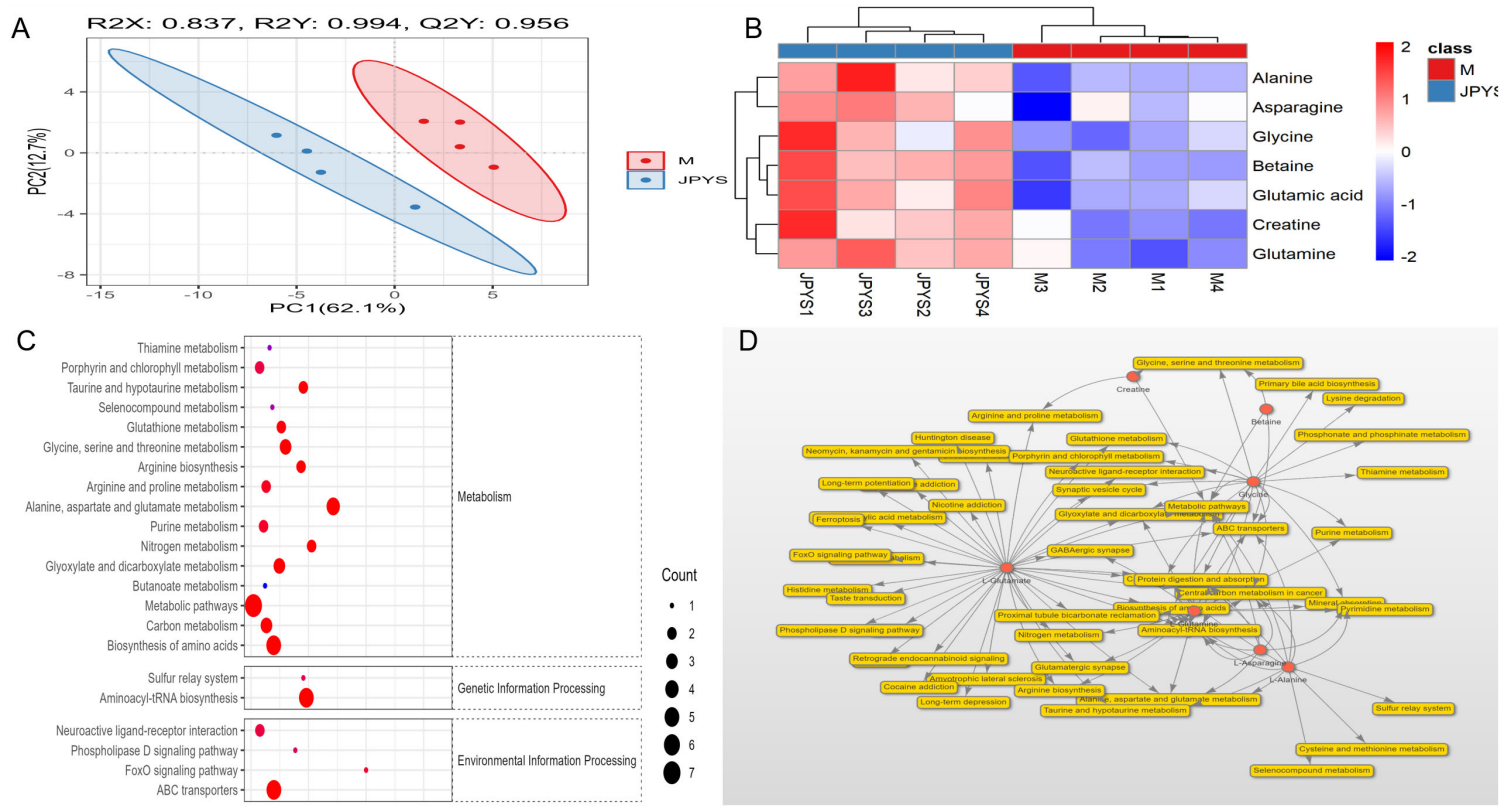
The metabolomic analysis in kidney tissue to further investigate the metabolic between JPYS rats and CKD rats. **(A)** The PLSDA indicated a distinct separation between JPY rats and CKD rats. **(B)** An examination of renal metabolism post-JPYS intervention identified seven metabolites that exhibited significantly elevated levels. **(C)** The identification of KEGG pathways associated with the differential metabolic functions observed between the two groups. **(D)** The interactive network graph analysis indicated that Betaine plays a regulatory role in Glycine, Serine, and Threonine metabolism, metabolic pathways, and ABC transporters.

To identify metabolic pathways, we conducted metabolomic in kidney sample to further investigate the metabolic between JPYS rats and CKD rats. The Partial Least Squares Discriminant Analysis (PLSDA) conducted in the field of metabolomics indicates a distinct separation between JPY rats and CKD rats, as illustrated in **Figure 6A**. An examination of renal metabolism post-JPYS intervention identified seven metabolites that exhibited significantly elevated levels, namely Betaine, Glycine, Alanine, Asparagine, Glutamic acid, Creatine, and Glutamine, as depicted in **Figure 6B**. The identification of KEGG pathways associated with the differential metabolic functions observed between the two groups (**Figure 6C**). The relevant differential pathways encompassed Glycine, Serine, and Threonine metabolism, as well as amino acid biosynthesis, aligning with the results obtained from RNA sequencing. Additionally, an interactive network graph analysis indicated that Betaine plays a regulatory role in Glycine, Serine, and Threonine metabolism, metabolic pathways, and ABC transporters (**Figure 6D**).

**Figure 7:**
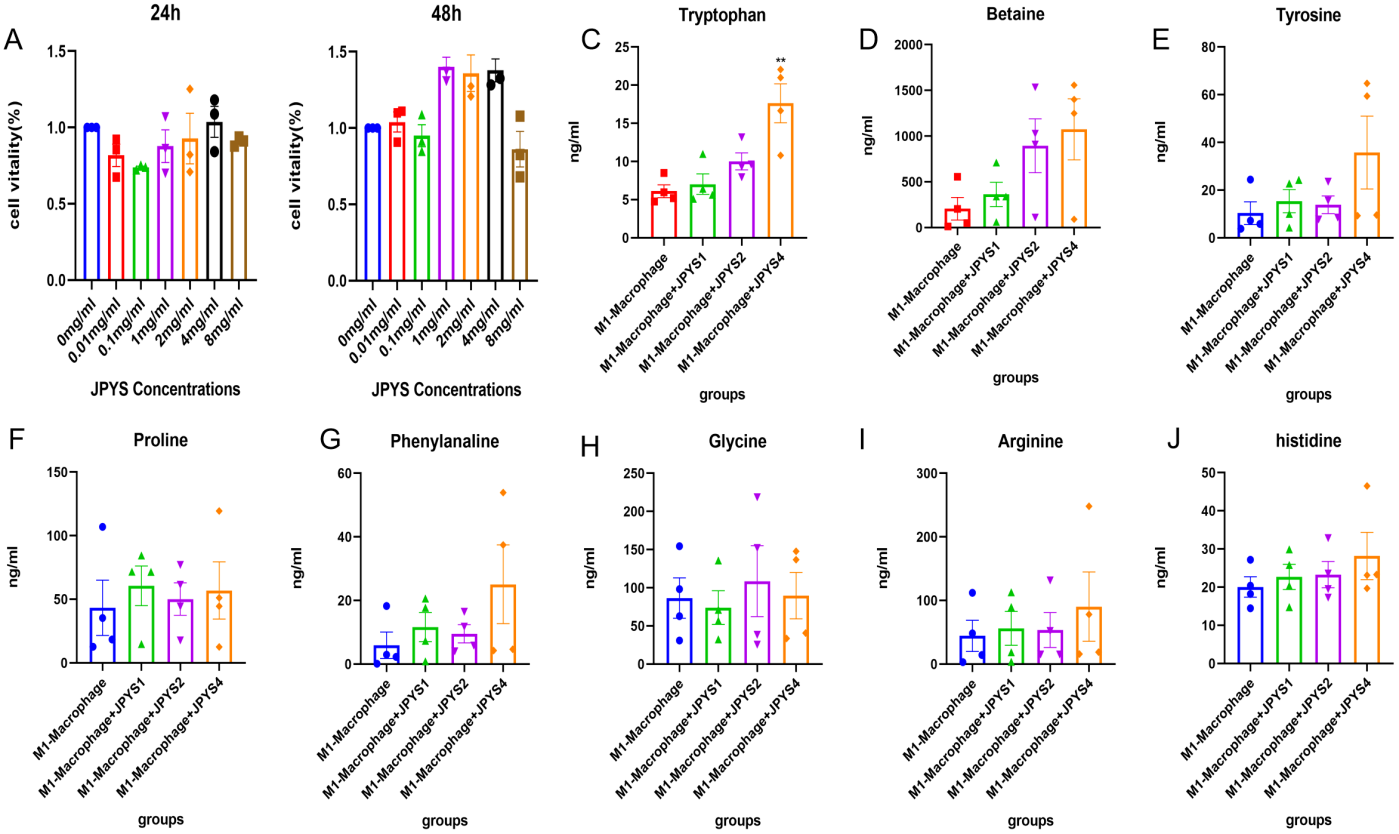
Metabolic pathway changed in M1 macrophage polarization incubated with JPYS. **(A, B)** THP-1 cells and incubated into macrophages, followed by incubated in different doses of JPYS for 24h and 48h. **(C-J)** Changes in amino acid metabolic pathways in M1 macrophage polarization incubated with JPYS, particularly the tryptophan was up-regulated after treatment with JPYS. **Represents a significant variation compare with the CKD group among the multiple comparisons. **P < 0.05.

**Figure 8:**
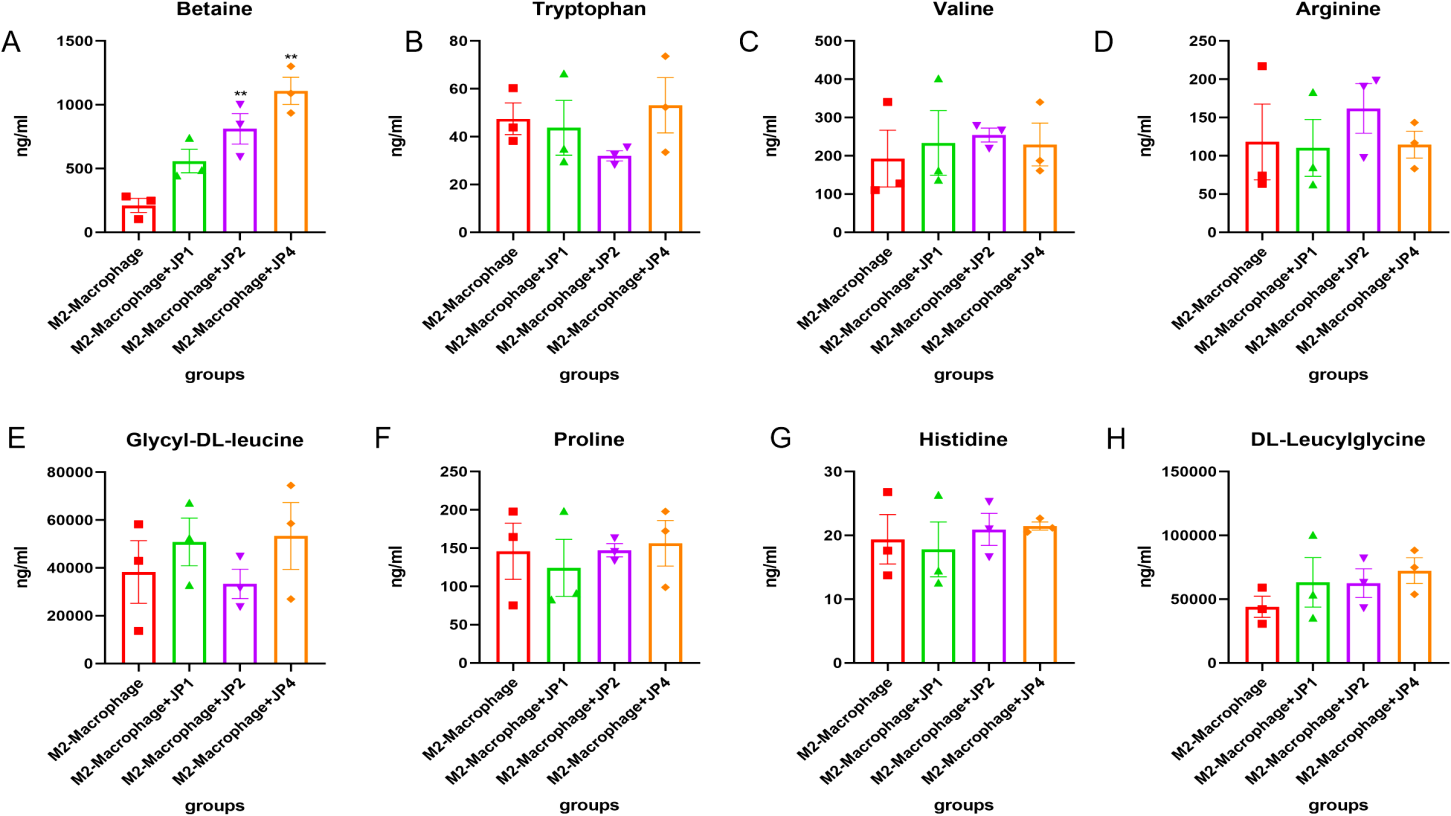
Metabolic pathway changed in M2 macrophage polarization incubated with JPYS. **(A-H)** metabolic pathways particularly Betaine was up-regulated after treatment with JPYS. **Represents a significant variation compare with the CKD group among the multiple comparisons. **P < 0.05.

To confirm the amino acid metabolism in macrophages, we utilized THP-1 cells and incubated into macrophages, followed by stimulation to generate M1 and M2 polarization macrophages for further metabolic analysis. In CCK-8, we observed that 1mg/ml, 2mg/ml and 4mg/ml shows positive influence in THP-1 cells while 8mg/ml JPYS formula downregulated THP-1 proliferation (**Figures 7A, B**). With the macrophage UPLC-MS/MS, we observed changes in amino acid metabolic pathways (**Figures 7**, **8**). The tryptophan was up-regulated after treatment with JPYS formula (**Figure 7C**). And betaine was up-regulated after treatment with JPYS formula (**Figure 8A**).

## 4 Discussion

In previous research, the JPYS formula has been shown to have convincing effects in anti-inflammation, anti-fibrosis, and the restoration of iron metabolism in CKD rats (14, 25). However, the underlying mechanisms by which the JPYS formula inhibits inflammation, especially via macrophage activity, remain unclear.

In the study, we initially employed network pharmacology to predict the active ingredients of the JPYS formula that are pharmacologically effective in CKD. This was followed by the construction and analysis of protein interaction networks, as well as conducting GO and KEGG enrichment analyses. Within the enriched pathways, we identified macrophage polarization as a potential target pathway of the JPYS formula, in relation to inflammatory injuries associated with CKD. This is consistent with the inflammatory injury that occurs during the natural progression of CKD. In line with this, in the *in vivo* model of CKD, the JPYS formula was shown to improve kidney function and alleviate kidney histopathological inflammatory damage. To validate the network pharmacology results, we looked further into macrophage immunophenotype expression and found that CKD rats had higher levels of CD68, CD86, and CD206, while the JPYS formula may have caused downregulation of macrophage surface marker expression in CKD. Furthermore, transcriptomic profiling of kidney tissue has indicated that the metabolic pathways linked to “Glycine, serine, and threonine metabolism” and “Biosynthesis of amino acids” are enriched as potential pathways of interest from, through which the JPYS formula provides renoprotection.

We then carried out additional validation using kidney proteomics, which identified the “metabolism” and “immune system” pathways as important mechanisms via which the JPYS formula has its therapeutic effects in CKD. The analyses also identified cellular functions, such as the regulation of the actin cytoskeleton, phagosomes, and lysosomes, in addition to significant associations with macrophages, which play a pivotal role in immune function. Additionally, tryptophan was found to be essential for the metabolism pathway. This finding is consistent with the network pharmacology analysis, indicating that macrophages may serve as important therapeutic targets in the treatment of CKD with JPYS.

Building further on this hypothesis, the metabolomics analysis from kidney tissue showed that significant differential pathways, such as the metabolism of glycine, serine, and threonine as well as the biosynthesis of amino acids, were enriched after JPYS intervention. Additionally, we observed a significant overexpression of betaine in the kidney tissue, which is probably related to the inclusion of Astragalus membranaceus and Cistanche deserticola (26, 27). Enrichment of the pathways related to glycine, serine, and threonine metabolism appear to correlate with the increased levels of betaine. Macrophage polarization was also impacted by the JPYS intervention, according to data from the *in vitro* studies. In particular, treatment with the JPYS formula increased tryptophan levels in the context of M1 macrophage polarization. JPYS formula treatment also increased the expression of betaine during the process of M2 macrophage polarization. Together, the JPYS formula may have protective effects against CKD injury by reducing inflammatory damage through the interaction of macrophage polarization and amino acid metabolism.

Macrophages are essential parts of kidney tissue which play critical roles in renal inflammation, the immune response, and the maintenance of kidney homeostasis (28, 29). In CKD, the persistent activation of pro-inflammatory monocytes and the presence of reparative macrophages contribute to conditions like glomerulosclerosis and tubulointerstitial fibrosis (30). Macrophage polarization, characterized by M1 pro-inflammatory and M2 reparative phenotypes, is a response to inflammatory stimuli, and the transition from M1 to M2 macrophages has been observed during the progression of CKD (31). Although certain herbal ingredients have demonstrated potential in regulating macrophage polarization and lowering inflammation, further research is needed to fully understand the impact of herbal treatments on aberrant macrophage-driven inflammation (32).

Current research indicates that the JPYS formula has the ability to influence both M1 and M2 macrophage polarization. Considering the role of amino acids, tryptophan metabolism can be improved by the JPYS formula in the context of M1 macrophage polarization. As an essential aromatic amino acid, tryptophan plays a key role in cellular synthesis, homeostasis maintenance, and it has been implicated in CKD progression (33). Disturbances in tryptophan metabolism are frequently reported in CKD patients, leading to worsening renal fibrosis and the progression of CKD, by causing metabolites to activate the aryl hydrocarbon receptor. As CKD advances, uremic toxins accumulate due to inadequate renal excretion, further resulting in deterioration of the condition (34). Conversely, disturbances in tryptophan metabolism can affect the kynurenine pathway, influencing the production of serotonin, indole-pyruvate derivatives, and tryptamine (34).

This study’s findings indicate that the JPYS formula offers protective effects against inflammation-induced damage driven by M1 macrophages. This therapeutic effect may be due to the modulation of tryptophan levels by JPYS. Moreover, it has been shown that the JPYS formula raises levels of betaine, which is a neutral amino acid derivative that is associated with maintaining organ homeostasis and halting the progression of disease. Previous studies have demonstrated the betaine in lowering steatosis, inflammation, and fibrosis in metabolism-associated fatty liver disease as well as oxidative stress and inflammation linked to alcoholic liver disease. Among other benefits, betaine has also been demonstrated to maintain the integrity of the intestinal epithelial barrier, control adipose function, and prevent the development of cancer (35–37). In the context of kidney health, betaine plays a crucial role in protecting cells against osmotic stress, exhibiting anti-inflammatory and antioxidant properties. Furthermore, low betaine levels have been linked to increased intestinal dysbiosis, oxidative stress, inflammation, and kidney damage, underscoring its significance as a metabolite for assessing the stages of CKD (38, 39). Therefore, patients with CKD may benefit from incorporating betaine-rich diets into their diets. Together, the available evidence generally supports using the JPYS formula as an effective modulator of amino acid metabolism during macrophage polarization.

One previous study demonstrated that amino acids play a significant role in modulating the inflammatory resolution processes, particularly through their interaction with macrophages, specifically in terms of polarization and secretion (40). In the current study’s enrichment analyses, the “Glycine, Serine, and Threonine Metabolism” pathway (KEGG map 00260) was shown to play a crucial role in the mechanism of action in JPYS treatment by regulating immunity and mitigating inflammatory damage. Furthermore, betaine in JPYS promotes the upregulation of glycine, which further enhances the expression of serine and threonine. Elevated levels of serine also facilitate tryptophan metabolism. Finally, the activation of amino acid metabolism, particularly the glycine, serine, and threonine metabolism pathways, regulates macrophage polarization and ultimately alleviates renal immune and inflammatory damage.

Treatment with JPYS, which stands for “strengthening the spleen and kidney,” is a traditional approach in Chinese medicine that aims to enhance blood circulation, eliminate dampness, and detoxify the body (41, 42). The JPYS formula has been widely applied in clinical settings and previous research has highlighted its various effects in delaying CKD progression. However, there is a research gap regarding the impact of the JPYS formula on immune function, which is crucial in understanding its potential therapeutic effects.

This study employed network pharmacology, RNA-seq, proteomic and metabolic analysis both *in vivo* and *in vitro.* We hypothesize that the JPYS formula elevates betaine levels in the kidney, thereby impacting amino acid synthesis and metabolism, particularly in pathways related to glycine, serine, and threonine metabolism. Ultimately, this modulation appears to influence macrophage polarization, which may represent a potential target for the JPYS formula in order to mitigate inflammatory injury and provide protection against CKD. Additionally, the study explored the formula’s role in immune regulation, inflammation modulation, in macrophage polarization, and its impact on metabolic changes to inhibit the progression of CKD.

## 5 Conclusion

Taken together, our findings suggest that the JPYS formula exerts its therapeutic effects through multiple mechanisms. These mechanisms include modulating inflammation, immune response, and macrophage polarization, as well as influencing metabolic changes. The interaction between amino acid metabolism and polarization, specifically the involvement of tryptophan in M1 polarization and betaine in M2 polarization, is a crucial mechanism of the JPYS formula in reducing inflammatory damage in CKD and decelerating its progression.

## Data Availability

The datasets presented in this study can be found in online repositories. The names of the repository/repositories and accession number(s) can be found below: https://www.ncbi.nlm.nih.gov/, bioproject/1090638 and www.iprox.cn. ID: IPX0010760000.
