# Supplementary material for: Network pharmacology and multi-omics validation of the Jianpi-Yishen formula in the treatment of chronic kidney disease
Source: Front Immunol. 2025 Jan 14;15:1512519. doi: 10.3389/fimmu.2024.1512519 (PMC11772200; doi:10.3389/fimmu.2024.1512519)
Supplement: Supplementary file 1 [file Table1.docx]

| Name | Company | Number |
| --- | --- | --- |
| CD68 | Servicebio | GB11067-100 |
| CD86 | Novus | NBP2-25208 |
| CD206 | Cell Signaling Technology | 24595S |
| PMA | Beyotime | S1819 |
| LPS | Merck | L6529 |
| IFN-R | proteintech | 102227 |

Table1

Table 2.Screening of active ingredients in Jianpi Yishen formula

| Herbal Name |  | Molecular ID | Molecular Name | Bioavailability/% | Drug like |
| --- | --- | --- | --- | --- | --- |
| Astragali Radix/Glycyrrhizae Radix et Rhizoma Praeparata cum Melle |  | MOL000211 | Mairin | 55.38 | 0.78 |
| Astragali Radix/Glycyrrhizae Radix et Rhizoma Praeparata cum Melle/Amomi Fructus Rotundus |  | MOL000239 | Jaranol | 50.83 | 0.29 |
| Astragali Radix/Atractylodis Macrocephalae Rhizoma |  | MOL000033 | (3S,8S,9S,10R,13R,14S,17R)-10,13-dimethyl-17-[(2R,5S)-5-propan-2-yloctan-2-yl]-2,3,4,7,8,9,11,12,14,15,16,17-dodecahydro-1H-cyclopenta[a]phenanthren-3-ol | 36.23 | 0.78 |
| Astragali Radix/Glycyrrhizae Radix et Rhizoma Praeparata cum Melle |  | MOL000354 | isorhamnetin | 49.60 | 0.31 |
|  |  | MOL000417 | Calycosin | 47.75 | 0.24 |
|  |  | MOL000422 | kaempferol | 41.88 | 0.24 |
|  |  | MOL000392 | formononetin | 69.67 | 0.21 |
| Astragali Radix/Cistanches Herba/Amomi Fructus Rotundus/Glycyrrhizae Radix et Rhizoma Praeparata cum Melle |  | MOL000098 | quercetin | 46.43 | 0.28 |
| Cistanches Herba/Rhei Radix et Rhizoma |  | MOL000358 | beta-sitosterol | 36.91 | 0.75 |
| Amomi Fructus Rotundus/Salviae Miltiorrhizae Radix et Rhizoma |  | MOL000006 | luteolin | 36.16 | 0.25 |
| Astragali Radix |  | MOL000296 | hederagenin | 36.91 | 0.75 |
|  |  | MOL000371 | 3,9-di-O-methylnissolin | 53.74 | 0.48 |
|  |  | MOL000374 | 5'-hydroxyiso-muronulatol-2',5'-di-O-glucoside | 41.72 | 0.69 |
|  |  | MOL000378 | 7-O-methylisomucronulatol | 74.69 | 0.30 |
|  |  | MOL000379 | 9,10-dimethoxypterocarpan-3-O-β-D-glucoside | 36.74 | 0.92 |
|  |  | MOL000380 | (6aR,11aR)-9,10-dimethoxy-6a,11a-dihydro-6H-benzofurano[3,2-c]chromen-3-ol | 64.26 | 0.42 |
|  |  | MOL000387 | Bifendate | 31.10 | 0.67 |
|  |  | MOL000398 | isoflavanone | 109.99 | 0.30 |
|  |  | MOL000433 | FA | 68.96 | 0.71 |
|  |  | MOL000438 | (3R)-3-(2-hydroxy-3,4-dimethoxyphenyl)chroman-7-ol | 67.67 | 0.26 |
|  |  | MOL000439 | isomucronulatol-7,2'-di-O-glucosiole | 49.28 | 0.62 |
|  |  | MOL000442 | 1,7-Dihydroxy-3,9-dimethoxy pterocarpene | 39.05 | 0.48 |
| Atractylodis Macrocephalae Rhizoma |  | MOL000018 | (+/-)-Isoborneol | 86.98 | 0.05 |
|  |  | MOL000020 | 12-senecioyl-2E,8E,10E-atractylentriol | 62.40 | 0.22 |
|  |  | MOL000021 | 14-acetyl-12-senecioyl-2E,8E,10E-atractylentriol | 60.31 | 0.31 |
|  |  | MOL000022 | 14-acetyl-12-senecioyl-2E,8Z,10E-atractylentriol | 63.37 | 0.30 |
|  |  | MOL000028 | 汐-Amyrin | 39.51 | 0.76 |
|  |  | MOL000049 | 3汕-acetoxyatractylone | 54.07 | 0.22 |
|  |  | MOL000072 | 8汕-ethoxy atractylenolide ｋ | 35.95 | 0.21 |
| Dioscoreae Rhizoma |  | MOL000136 | Dioscoreside C | 10.32 | 0.03 |
|  |  | MOL001559 | piperlonguminine | 30.71 | 0.18 |
|  |  | MOL001736 | (-)-taxifolin | 60.51 | 0.27 |
|  |  | MOL000310 | Denudatin B | 61.47 | 0.38 |
|  |  | MOL000322 | Kadsurenone | 54.72 | 0.38 |
|  |  | MOL005429 | hancinol | 64.01 | 0.37 |
|  |  | MOL005430 | hancinone C | 59.05 | 0.39 |
|  |  | MOL005435 | 24-Methylcholest-5-enyl-3belta-O-glucopyranoside_qt | 37.58 | 0.72 |
|  |  | MOL005438 | campesterol | 37.58 | 0.71 |
|  |  | MOL005440 | Isofucosterol | 43.78 | 0.76 |
|  |  | MOL000449 | Stigmasterol | 43.83 | 0.76 |
|  |  | MOL005458 | Dioscoreside C_qt | 36.38 | 0.87 |
|  |  | MOL000546 | diosgenin | 80.88 | 0.81 |
|  |  | MOL005461 | Doradexanthin | 38.16 | 0.54 |
|  |  | MOL005463 | Methylcimicifugoside_qt | 31.69 | 0.24 |
|  |  | MOL005465 | AIDS180907 | 45.33 | 0.77 |
|  |  | MOL000953 | CLR | 37.87 | 0.68 |
| Cistanches Herba |  | MOL005320 | arachidonate | 45.57 | 0.20 |
|  |  | MOL005384 | suchilactone | 57.52 | 0.56 |
|  |  | MOL007563 | Yangambin | 57.53 | 0.81 |
|  |  | MOL008871 | Marckine | 37.05 | 0.69 |
| Amomi Fructus Rotundus |  | MOL000224 | (4E,6E)-1,7-bis(3,4-dihydroxyphenyl)hepta-4,6-dien-3-one | 33.06 | 0.31 |
|  |  | MOL000228 | (2R)-7-hydroxy-5-methoxy-2-phenylchroman-4-one | 55.23 | 0.20 |
|  |  | MOL000230 | Pinocembrin | 57.56 | 0.20 |
|  |  | MOL000235 | 1,7-diphenyl-3,5-dihydroxy-1-heptene | 49.01 | 0.18 |
|  |  | MOL000238 | 1,7-diphenyl-5-hydroxy-6-hepten-3-one | 32.65 | 0.18 |
|  |  | MOL000242 | 7-O-Methyleriodictyol | 56.56 | 0.27 |
|  |  | MOL000243 | alpinolide peroxide | 87.67 | 0.19 |
|  |  | MOL000258 | dehydrodiisoeugenol | 56.84 | 0.29 |
|  |  | MOL000260 | 5-[(2R,3R)-7-methoxy-3-methyl-5-[(E)-prop-1-enyl]-2,3-dihydrobenzofuran-2-yl]-1,3-benzodioxole | 65.55 | 0.40 |
| Salviae Miltiorrhizae Radix et Rhizoma |  | MOL001601 | 1,2,5,6-tetrahydrotanshinone | 38.75 | 0.36 |
|  |  | MOL001659 | Poriferasterol | 43.83 | 0.76 |
|  |  | MOL001771 | poriferast-5-en-3beta-ol | 36.91 | 0.75 |
|  |  | MOL001942 | isoimperatorin | 45.46 | 0.23 |
|  |  | MOL002222 | sugiol | 36.11 | 0.28 |
|  |  | MOL002651 | Dehydrotanshinone II A | 43.76 | 0.40 |
|  |  | MOL002776 | Baicalin | 40.12 | 0.75 |
|  |  | MOL000569 | digallate | 61.85 | 0.26 |
|  |  | MOL006824 | 汐-amyrin | 39.51 | 0.76 |
|  |  | MOL007036 | 5,6-dihydroxy-7-isopropyl-1,1-dimethyl-2,3-dihydrophenanthren-4-one | 33.77 | 0.29 |
|  |  | MOL007041 | 2-isopropyl-8-methylphenanthrene-3,4-dione | 40.86 | 0.23 |
|  |  | MOL007045 | 3汐-hydroxytanshinoneｊa | 44.93 | 0.44 |
|  |  | MOL007048 | (E)-3-[2-(3,4-dihydroxyphenyl)-7-hydroxy-benzofuran-4-yl]acrylic acid | 48.24 | 0.31 |
|  |  | MOL007049 | 4-methylenemiltirone | 34.35 | 0.23 |
|  |  | MOL007050 | 2-(4-hydroxy-3-methoxyphenyl)-5-(3-hydroxypropyl)-7-methoxy-3-benzofurancarboxaldehyde | 62.78 | 0.40 |
|  |  | MOL007051 | 6-o-syringyl-8-o-acetyl shanzhiside methyl ester | 46.69 | 0.71 |
|  |  | MOL007058 | formyltanshinone | 73.44 | 0.42 |
|  |  | MOL007059 | 3-beta-Hydroxymethyllenetanshiquinone | 32.16 | 0.41 |
|  |  | MOL007061 | Methylenetanshinquinone | 37.07 | 0.36 |
|  |  | MOL007063 | przewalskin a | 37.11 | 0.65 |
|  |  | MOL007064 | przewalskin b | 110.32 | 0.44 |
|  |  | MOL007068 | Przewaquinone B | 62.24 | 0.41 |
|  |  | MOL007069 | przewaquinone c | 55.74 | 0.40 |
|  |  | MOL007070 | (6S,7R)-6,7-dihydroxy-1,6-dimethyl-8,9-dihydro-7H-naphtho[8,7-g]benzofuran-10,11-dione | 41.31 | 0.45 |
|  |  | MOL007071 | przewaquinone f | 40.31 | 0.46 |
|  |  | MOL007077 | sclareol | 43.67 | 0.21 |
|  |  | MOL007079 | tanshinaldehyde | 52.47 | 0.45 |
|  |  | MOL007081 | Danshenol B | 57.95 | 0.56 |
|  |  | MOL007082 | Danshenol A | 56.97 | 0.52 |
|  |  | MOL007085 | Salvilenone | 30.38 | 0.38 |
|  |  | MOL007088 | cryptotanshinone | 52.34 | 0.40 |
|  |  | MOL007093 | dan-shexinkum d | 38.88 | 0.55 |
|  |  | MOL007094 | danshenspiroketallactone | 50.43 | 0.31 |
|  |  | MOL007098 | deoxyneocryptotanshinone | 49.40 | 0.29 |
|  |  | MOL007100 | dihydrotanshinlactone | 38.68 | 0.32 |
|  |  | MOL007101 | dihydrotanshinoneｉ | 45.04 | 0.36 |
|  |  | MOL007105 | epidanshenspiroketallactone | 68.27 | 0.31 |
|  |  | MOL007107 | C09092 | 36.07 | 0.25 |
|  |  | MOL007108 | isocryptotanshi-none | 54.98 | 0.39 |
|  |  | MOL007111 | Isotanshinone II | 49.92 | 0.40 |
|  |  | MOL007115 | manool | 45.04 | 0.20 |
|  |  | MOL007118 | microstegiol | 39.61 | 0.28 |
|  |  | MOL007119 | miltionone ｉ | 49.68 | 0.32 |
|  |  | MOL007120 | miltionone ｊ | 71.03 | 0.44 |
|  |  | MOL007121 | miltipolone | 36.56 | 0.37 |
|  |  | MOL007122 | Miltirone | 38.76 | 0.25 |
|  |  | MOL007123 | miltirone ｊ | 44.95 | 0.24 |
|  |  | MOL007124 | neocryptotanshinone ii | 39.46 | 0.23 |
|  |  | MOL007125 | neocryptotanshinone | 52.49 | 0.32 |
|  |  | MOL007127 | 1-methyl-8,9-dihydro-7H-naphtho[5,6-g]benzofuran-6,10,11-trione | 34.72 | 0.37 |
|  |  | MOL007130 | prolithospermic acid | 64.37 | 0.31 |
|  |  | MOL007132 | (2R)-3-(3,4-dihydroxyphenyl)-2-[(Z)-3-(3,4-dihydroxyphenyl)acryloyl]oxy-propionic acid | 109.38 | 0.35 |
|  |  | MOL007140 | (Z)-3-[2-[(E)-2-(3,4-dihydroxyphenyl)vinyl]-3,4-dihydroxy-phenyl]acrylic acid | 88.54 | 0.26 |
|  |  | MOL007141 | salvianolic acid g | 45.56 | 0.61 |
|  |  | MOL007142 | salvianolic acid j | 43.38 | 0.72 |
|  |  | MOL007143 | salvilenone ｉ | 32.43 | 0.23 |
|  |  | MOL007145 | salviolone | 31.72 | 0.24 |
|  |  | MOL007149 | NSC 122421 | 34.49 | 0.28 |
|  |  | MOL007150 | (6S)-6-hydroxy-1-methyl-6-methylol-8,9-dihydro-7H-naphtho[8,7-g]benzofuran-10,11-quinone | 75.39 | 0.46 |
|  |  | MOL007151 | Tanshindiol B | 42.67 | 0.45 |
|  |  | MOL007152 | Przewaquinone E | 42.85 | 0.45 |
|  |  | MOL007154 | tanshinone iia | 49.89 | 0.40 |
|  |  | MOL007155 | (6S)-6-(hydroxymethyl)-1,6-dimethyl-8,9-dihydro-7H-naphtho[8,7-g]benzofuran-10,11-dione | 65.26 | 0.45 |
|  |  | MOL007156 | tanshinone ｎ | 45.64 | 0.30 |
| Rhei Radix et Rhizoma |  | MOL001237 | o-Acetyltoluene | 38.96 | 0.02 |
|  |  | MOL002235 | EUPATIN | 50.80 | 0.41 |
|  |  | MOL002251 | Mutatochrome | 48.64 | 0.61 |
|  |  | MOL002259 | Physciondiglucoside | 41.65 | 0.63 |
|  |  | MOL002260 | Procyanidin B-5,3'-O-gallate | 31.99 | 0.32 |
|  |  | MOL002268 | rhein | 47.07 | 0.28 |
|  |  | MOL002276 | Sennoside E_qt | 50.69 | 0.61 |
|  |  | MOL002280 | Torachrysone-8-O-beta-D-(6'-oxayl)-glucoside | 43.02 | 0.74 |
|  |  | MOL002281 | Toralactone | 46.46 | 0.24 |
|  |  | MOL002288 | Emodin-1-O-beta-D-glucopyranoside | 44.81 | 0.80 |
|  |  | MOL002293 | Sennoside D_qt | 61.06 | 0.61 |
|  |  | MOL002297 | Daucosterol_qt | 35.89 | 0.70 |
|  |  | MOL002303 | palmidin A | 32.45 | 0.65 |
|  |  | MOL000471 | aloe-emodin | 83.38 | 0.24 |
|  |  | MOL000554 | gallic acid-3-O-(6'-O-galloyl)-glucoside | 30.25 | 0.67 |
|  |  | MOL000096 | (-)-catechin | 49.68 | 0.24 |
| Glycyrrhizae Radix et Rhizoma Praeparata cum Melle |  | MOL001484 | Inermine | 75.18 | 0.54 |
|  |  | MOL001792 | DFV | 32.76 | 0.18 |
|  |  | MOL002311 | Glycyrol | 90.78 | 0.67 |
|  |  | MOL002565 | Medicarpin | 49.22 | 0.34 |
|  |  | MOL000359 | sitosterol | 36.91 | 0.75 |
|  |  | MOL003656 | Lupiwighteone | 51.64 | 0.37 |
|  |  | MOL003896 | 7-Methoxy-2-methyl isoflavone | 42.56 | 0.20 |
|  |  | MOL004328 | naringenin | 59.29 | 0.21 |
|  |  | MOL004805 | (2S)-2-[4-hydroxy-3-(3-methylbut-2-enyl)phenyl]-8,8-dimethyl-2,3-dihydropyrano[2,3-f]chromen-4-one | 31.79 | 0.72 |
|  |  | MOL004806 | euchrenone | 30.29 | 0.57 |
|  |  | MOL004808 | glyasperin B | 65.22 | 0.44 |
|  |  | MOL004810 | glyasperin F | 75.84 | 0.54 |
|  |  | MOL004811 | Glyasperin C | 45.56 | 0.40 |
|  |  | MOL004814 | Isotrifoliol | 31.94 | 0.42 |
|  |  | MOL004815 | (E)-1-(2,4-dihydroxyphenyl)-3-(2,2-dimethylchromen-6-yl)prop-2-en-1-one | 39.62 | 0.35 |
|  |  | MOL004820 | kanzonols W | 50.48 | 0.52 |
|  |  | MOL004824 | (2S)-6-(2,4-dihydroxyphenyl)-2-(2-hydroxypropan-2-yl)-4-methoxy-2,3-dihydrofuro[3,2-g]chromen-7-one | 60.25 | 0.63 |
|  |  | MOL004827 | Semilicoisoflavone B | 48.78 | 0.55 |
|  |  | MOL004828 | Glepidotin A | 44.72 | 0.35 |
|  |  | MOL004829 | Glepidotin B | 64.46 | 0.34 |
|  |  | MOL004833 | Phaseolinisoflavan | 32.01 | 0.45 |
|  |  | MOL004835 | Glypallichalcone | 61.60 | 0.19 |
|  |  | MOL004838 | 8-(6-hydroxy-2-benzofuranyl)-2,2-dimethyl-5-chromenol | 58.44 | 0.38 |
|  |  | MOL004841 | Licochalcone B | 76.76 | 0.19 |
|  |  | MOL004848 | licochalcone G | 49.25 | 0.32 |
|  |  | MOL004849 | 3-(2,4-dihydroxyphenyl)-8-(1,1-dimethylprop-2-enyl)-7-hydroxy-5-methoxy-coumarin | 59.62 | 0.43 |
|  |  | MOL004855 | Licoricone | 63.58 | 0.47 |
|  |  | MOL004856 | Gancaonin A | 51.08 | 0.40 |
|  |  | MOL004857 | Gancaonin B | 48.79 | 0.45 |
|  |  | MOL004860 | licorice glycoside E | 32.89 | 0.27 |
|  |  | MOL004863 | 3-(3,4-dihydroxyphenyl)-5,7-dihydroxy-8-(3-methylbut-2-enyl)chromone | 66.37 | 0.41 |
|  |  | MOL004864 | 5,7-dihydroxy-3-(4-methoxyphenyl)-8-(3-methylbut-2-enyl)chromone | 30.49 | 0.41 |
|  |  | MOL004866 | 2-(3,4-dihydroxyphenyl)-5,7-dihydroxy-6-(3-methylbut-2-enyl)chromone | 44.15 | 0.41 |
|  |  | MOL004879 | Glycyrin | 52.61 | 0.47 |
|  |  | MOL004882 | Licocoumarone | 33.21 | 0.36 |
|  |  | MOL004883 | Licoisoflavone | 41.61 | 0.42 |
|  |  | MOL004884 | Licoisoflavone B | 38.93 | 0.55 |
|  |  | MOL004885 | licoisoflavanone | 52.47 | 0.54 |
|  |  | MOL004891 | shinpterocarpin | 80.30 | 0.73 |
|  |  | MOL004898 | (E)-3-[3,4-dihydroxy-5-(3-methylbut-2-enyl)phenyl]-1-(2,4-dihydroxyphenyl)prop-2-en-1-one | 46.27 | 0.31 |
|  |  | MOL004903 | liquiritin | 65.69 | 0.74 |
|  |  | MOL004904 | licopyranocoumarin | 80.36 | 0.65 |
|  |  | MOL004905 | 3,22-Dihydroxy-11-oxo-delta(12)-oleanene-27-alpha-methoxycarbonyl-29-oic acid | 34.32 | 0.55 |
|  |  | MOL004907 | Glyzaglabrin | 61.07 | 0.35 |
|  |  | MOL004908 | Glabridin | 53.25 | 0.47 |
|  |  | MOL004910 | Glabranin | 52.90 | 0.31 |
|  |  | MOL004911 | Glabrene | 46.27 | 0.44 |
|  |  | MOL004912 | Glabrone | 52.51 | 0.50 |
|  |  | MOL004913 | 1,3-dihydroxy-9-methoxy-6-benzofurano[3,2-c]chromenone | 48.14 | 0.43 |
|  |  | MOL004914 | 1,3-dihydroxy-8,9-dimethoxy-6-benzofurano[3,2-c]chromenone | 62.90 | 0.53 |
|  |  | MOL004915 | Eurycarpin A | 43.28 | 0.37 |
|  |  | MOL004917 | glycyroside | 37.25 | 0.79 |
|  |  | MOL004924 | (-)-Medicocarpin | 40.99 | 0.95 |
|  |  | MOL004935 | Sigmoidin-B | 34.88 | 0.41 |
|  |  | MOL004941 | (2R)-7-hydroxy-2-(4-hydroxyphenyl)chroman-4-one | 71.12 | 0.18 |
|  |  | MOL004945 | (2S)-7-hydroxy-2-(4-hydroxyphenyl)-8-(3-methylbut-2-enyl)chroman-4-one | 36.57 | 0.32 |
|  |  | MOL004948 | Isoglycyrol | 44.70 | 0.84 |
|  |  | MOL004949 | Isolicoflavonol | 45.17 | 0.42 |
|  |  | MOL004957 | HMO | 38.37 | 0.21 |
|  |  | MOL004959 | 1-Methoxyphaseollidin | 69.98 | 0.64 |
|  |  | MOL004961 | Quercetin der. | 46.45 | 0.33 |
|  |  | MOL004966 | 3'-Hydroxy-4'-O-Methylglabridin | 43.71 | 0.57 |
|  |  | MOL000497 | licochalcone a | 40.79 | 0.29 |
|  |  | MOL004974 | 3'-Methoxyglabridin | 46.16 | 0.57 |
|  |  | MOL004978 | 2-[(3R)-8,8-dimethyl-3,4-dihydro-2H-pyrano[6,5-f]chromen-3-yl]-5-methoxyphenol | 36.21 | 0.52 |
|  |  | MOL004980 | Inflacoumarin A | 39.71 | 0.33 |
|  |  | MOL004985 | icos-5-enoic acid | 30.70 | 0.20 |
|  |  | MOL004988 | Kanzonol F | 32.47 | 0.89 |
|  |  | MOL004989 | 6-prenylated eriodictyol | 39.22 | 0.41 |
|  |  | MOL004990 | 7,2',4'-trihydroxy－5-methoxy-3－arylcoumarin | 83.71 | 0.27 |
|  |  | MOL004991 | 7-Acetoxy-2-methylisoflavone | 38.92 | 0.26 |
|  |  | MOL004993 | 8-prenylated eriodictyol | 53.79 | 0.40 |
|  |  | MOL004996 | gadelaidic acid | 30.70 | 0.20 |
|  |  | MOL000500 | Vestitol | 74.66 | 0.21 |
|  |  | MOL005000 | Gancaonin G | 60.44 | 0.39 |
|  |  | MOL005001 | Gancaonin H | 50.10 | 0.78 |
|  |  | MOL005003 | Licoagrocarpin | 58.81 | 0.58 |
|  |  | MOL005007 | Glyasperins M | 72.67 | 0.59 |
|  |  | MOL005008 | Glycyrrhiza flavonol A | 41.28 | 0.60 |
|  |  | MOL005012 | Licoagroisoflavone | 57.28 | 0.49 |
|  |  | MOL005013 | 18α-hydroxyglycyrrhetic acid | 41.16 | 0.71 |
|  |  | MOL005016 | Odoratin | 49.95 | 0.30 |
|  |  | MOL005017 | Phaseol | 78.77 | 0.58 |
|  |  | MOL005018 | Xambioona | 54.85 | 0.87 |
|  |  | MOL005020 | dehydroglyasperins C | 53.82 | 0.37 |
